# Development of Metabolite-Responsive Transcription Factor Systems as Modular Platforms for Gene Expression Control

**DOI:** 10.3390/bios15120820

**Published:** 2025-12-18

**Authors:** Haekang Ji, Jiwon Lee, Kyeongseok Song, Yangwon Jeon, Geupil Jang, Youngdae Yoon

**Affiliations:** 1 Department of Environmental Health Science, Konkuk University, Seoul 05029, Republic of Korea; wl08140@konkuk.ac.kr (H.J.);; 2 Department of Energy Environmental and Chemical Engineering, Washington University in St. Louis, Saint Louis, MO 63130, USA; 3 School of Biological Sciences and Technology, Chonnam National University, Gwangju 61186, Republic of Korea

**Keywords:** transcription factors, MarR, TtgR, flavonoids, O-methyltransferase, synthetic biology

## Abstract

Traditional inducible systems typically induce the simultaneous expression of all genes controlled by similar promoters, thereby limiting their use. In this study, we used two metabolite-inducible systems, MarR from the *Escherichia coli mar* operon and TtgR from the *Pseudomonas putida ttg* operon, to assess their use as gene regulation platforms beyond reporter assays. Ligand-dependent transcription was validated using eGFP. The reporter was replaced with two flavonoid O-methyltransferases (OMTs), ROMT-9 and SOMT-2, under transcription factor (TF)-specific promoters. In *E. coli*, both systems enabled in using HPLC. TF-based expression did not impact enzyme activity. Induction with salicylic acid (MarR) produced stronger gains than that with 4′-hydroxyflavanone (TtgR), although the overall fold-changes in product levels were regulated by basal (leaky) expression. Thus, although transcriptional control was robust, enzymatic regulation was less stringent, highlighting the necessity for genetic engineering of components, including TFs, promoters, transcription factor binding sites, and ribosome binding sites, to reduce leakiness and expand the dynamic range. Overall, these orthogonal and modular TF-based systems offer a framework for independent and inducible control of multiple genes, with potential applications in biosensing, metabolic engineering, and programmable pathway design.

## 1. Introduction

Transcription factor (TF)-based biosensors are powerful tools in genetic engineering and synthetic biology that detect specific small molecules and translate these signals into measurable outputs via reporter genes, such as fluorescent proteins or enzymes [1,2]. The output intensity is typically proportional to ligand concentration, facilitating quantitative and real-time monitoring. TFs that respond to specific ligands have been used as genetic systems in biosensors [3,4,5]. In addition to detection, TF-based biosensors can function as ligand-inducible gene expression systems, enabling the temporal and conditional control of metabolic processes [6,7,8]. This control is particularly valuable in metabolic engineering, where the constitutive expression of pathway enzymes can impose a metabolic burden on host cells and reduce productivity.

Based on several previous studies, the performance of TF-based biosensors, including target selectivity, specificity, dynamic range, and detection limit, is determined by diverse factors, such as TF–ligand binding affinity, TF expression levels, and the strength of transcription factor binding sites (TFBSs) in both TF and reporter gene promoters [6,9,10]. Protein engineering of TFs and optimization of genetic elements, such as ribosome binding sites (RBSs) and TFBS sequences, enhance biosensor performance. Modulating the performance of biosensors ultimately relies on controlling the expression of regulatory proteins and reporter genes, as well as tuning their responsiveness; therefore, TF-based systems can be extended beyond sensing applications to serve as versatile platforms for gene regulation. Taken together, these characteristics highlight the potential of TF-based systems as modular and mutually orthogonal gene regulation platforms capable of operating independently without cross-interference between regulatory modules. This expands their utility far beyond conventional sensing, enabling programmable control of metabolic functions such as adaptive laboratory evolution, high-throughput screening, and synthetic pathway optimization [7,8,11,12].

Flavonoids are plant-derived natural compounds with diverse biological activities, including anticancer, anti-inflammatory, antifungal, and antibacterial properties [13,14]. They are also valued as dietary supplements owing to their beneficial effects on human health. Consequently, flavonoid biosynthesis has emerged as an important target in synthetic biology, often relying on the heterologous expression of multiple enzymes in engineered hosts [15,16,17]. Efficient flavonoid production requires precise regulation of pathway enzymes, as excessive accumulation of intermediates can be toxic or impose a metabolic burden. Therefore, it is critical to implement temporal control of enzyme expression to maximize the yield and maintain host cell viability. Various inducible and tunable gene expression systems have been developed to address this issue.

Traditional inducible systems, such as IPTG/LacI, arabinose/AraC, and tetracycline/TetR, have long been used to regulate transcription in microbial hosts [18,19]. These systems are well characterized and widely used, with tunability achieved through the modulation of promoter strength, ribosome-binding sites, and inducer concentrations. However, traditional inducible systems typically activate all genes under the same type of promoter simultaneously upon inducer addition, making it difficult to fine-tune or individually optimize the expression of multiple enzymes within a metabolic pathway [20]. Recent studies have attempted to improve these systems by combining dual-inducible elements for multistep biosynthetic pathways [21]; however, challenges such as leaky expression and a lack of orthogonality remain. Therefore, it is anticipated that the development of novel regulatory systems will further enhance efficiency by enabling more precise, flexible, and orthogonal control of enzyme expression. Ligand-responsive TF-based systems represent a promising alternative, as they enable tunable and independent regulation of multiple genes within the same cellular environment and provide temporal control over enzymatic steps in complex metabolic pathways [7,22,23].

In this study, we used two well-characterized bacterial transcriptional repressors MarR from the *Escherichia coli* marRAB operon, which responds to phenolic compounds, such as salicylate, and TtgR from the *Pseudomonas putida* ttgABC efflux operon, which responds to aromatic hydrocarbons, including resveratrol. Our previous study demonstrated that MarR- and TtgR-based *E. coli* biosensors can selectively detect salicylate and resveratrol, respectively, with improved specificity through protein engineering [24,25]. Therefore, we replaced the reporter gene with two flavonoid O-methyltransferase (OMT) genes, *SOMT-2* and *ROMT-9*, to establish orthogonal and ligand-inducible systems capable of directing flavonoid biotransformation into methylated derivatives. This design enables selective enzyme activation in response to cognate ligands and facilitates the comparative evaluation of substrate specificity and catalytic efficiency. Although background (leaky) expression and insufficient repression remain limitations of current systems, these challenges can be addressed through further optimization of TF components. Despite these limitations, the modularity, tunability, and orthogonality of TF-based systems highlight their potential as versatile platforms in metabolic engineering, biosensing, and synthetic biology. With continued refinement, they may provide powerful tools for the precise and programmable control of gene expression in complex metabolic networks, ultimately enabling more efficient pathway design and biosynthesis.

## 2. Materials and Methods

### 2.1. Materials

#### 2.1.1. Bacterial Strains and Plasmid Construction

*Escherichia coli* BL21 and DH5α strains were used as host cells for plasmid cloning and construction. For the mar system, a *marR*-deficient *E. coli* BL21 (DE3) strain (*E. coli*-marR) was used as the host. The genetic characteristics of the *E. coli* strains used in this study are listed in Table 1. Restriction enzymes (BamHI, NcoI, XhoI, and NdeI), DNA ligase, and DNA polymerase were purchased from New England Biolabs and Qiagen. The primers used for plasmid construction were synthesized by Macrogen (Seoul, Republic of Korea). The *SOMT-2* and *ROMT-9* genes were kindly provided by Dr. Joong-Hoon An (Konkuk University, Republic of Korea).

#### 2.1.2. Chemicals and Flavonoids

Flavonoids used in this study were purchased from Sigma-Aldrich (St. Louis, MO, USA) and Indofine Chemical Company (Hillsborough, NJ, USA). Salicylic acid (SA) was obtained from Sigma-Aldrich. A complete list of flavonoids, including their position-specific functional group substitutions, is provided in Table 2. The numbering for flavonoid structures is illustrated in Figure S1. All flavonoids were dissolved in DMSO to a final concentration of 50 mM and stored until use. All the other chemicals used in this study, including ethyl acetate, acetonitrile, formic acid, and degassed water, were of analytical grade.

### 2.2. Construction of Plasmids and E. coli Strains

Reporter plasmids carrying the fusion of *P_marR_::egfp* and *P_ttgABC_::egfp* and genes encoding MarR and TtgR were constructed in our previous studies [24,26]. The promoter regions of *mar* and *ttg* operon systems were amplified using PCR with appropriate primer pairs and inserted into the BglII/XbaI sites of pET-21 (a), then the gene encoding eGFP was inserted into BamHI/XhoI. The resulting plasmids were named pMar-eGFP and pTtg-eGFP. The regulatory plasmids carrying MarR and TtgR under the control of the T7 promoter were named pCDF-MarR and pCDF-TtgR, respectively. The plasmids carrying flavonoid-OMTs derived from soybean and rice, named SOMT-2 [27] and ROMT-9 [28], respectively, were kindly provided by Dr. Ahn (Konkuk University, Seoul, Republic of Korea) and were amplified using PCR with appropriate primer pairs. The genes were inserted into promoter-carrying plasmids containing BamHI/XhoI and BamHI/NdeI for SOMT-2 and ROMT-9, respectively.

For further investigation, *E. coli* strains for biosensors and biotransformation assays were generated by introducing a pair of plasmids corresponding to each TF system. Biosensor cells were generated by transforming the plasmid pairs of TFs and reporter plasmids, pCDF-MarRs/pMar-eGFP and pCDF-TtgRs/pTtg-eGFP. Similarly, plasmids carrying *P_marR_::OMTs* and *P_ttgABC_::OMTs* were co-transformed with plasmids carrying the corresponding regulatory proteins for the biotransformation assay. The plasmids and biosensor strains used in this study are listed in Table 1.

### 2.3. Biosensor Assay

To validate the regulation of gene expression in TF-based genetic systems, biosensors harboring pMar-eGFP/pCDF-MarR and pTtg-eGFP/pCDF-TtgR were used. Both biosensors were cultured overnight in lysogeny broth (LB) with appropriate antibiotics and then inoculated into fresh LB. Cells were grown until optical density absorbance at 600 nm (OD_600_) was 0.3–0.4 and exposed to compounds that induce biosensor systems, including salicylic acid and flavonoids. Additionally, an equal amount of DMSO was added to the control samples to eliminate solvent-related effects. The expression level of eGFP was measured over time for up to 24 h using a fluorescence plate reader (BioTek Synergy LX, Agilent Technologies, Santa Clara, CA, USA) at 480 nm excitation/510 nm emission wavelength. The responses toward inducers were indicated as arbitrary units of eGFP and compared with and without inducers. The measured values were converted to induction coefficient (I.C.) values, which were defined using the following formula with compensation for OD_600_ values:

(1) $$I.C.\mathrm{values}=\frac{\mathrm{arbitrary}\;\mathrm{unit}\;\mathrm{of}\;\mathrm{eGFP}}{\mathrm{arbitrary}\;\mathrm{unit}\;\mathrm{of}\;\mathrm{eGFP}\;\mathrm{at}\;0\;h}$$

### 2.4. Biotransformation of Flavonoids

The *E. coli* cells harboring pMar-OMTs (SOMT-2 and ROMT-9)/pCDF-MarRs and pTtg-OMTs/pCDF-TtgRs were investigated to validate their capability in TF-based genetic systems. Similar to the experimental conditions of the biosensor assay, the cells were cultured overnight in LB. The cells were inoculated into fresh LB and exposed to 0.5 mM SA for the *mar*-operon system and to either 0.05 mM 3′-FVA or 0.05 mM 4′-FVA for the *ttg*-operon system at 0.3 of OD_600_. After 2 h of further incubation, 1–5 mM of flavonoids was added and incubated for a designated time (up to 24 h) to measure the biotransformation efficiency. Cell cultures (1 mL) were extracted with 1.5 mL of ethyl acetate and dried under a speed vacuum dryer (NB503CIR; Global N Biotek Co., Ltd., Buchon-si, Republic of Korea). The extracts were dissolved in methanol (MeOH) and subjected to HPLC analysis.

### 2.5. HPLC Analysis

To evaluate the biotranformation efficiency by measuring the peak area of products, the extracts of flavonoids and their biotransformed products by OMTs that were dissolved in MeOH were analyzed using a YL9100 Plus HPLC system (Youngin Chromass Co., Ltd., Anyang, Republic of Korea) equipped with a ZORBAX Eclipse XDB-C18 reversed-phase column (4.6 × 150 mm; Agilent Technologies, Santa Clara, CA, USA) and photodiode array detector. Chromatographic separation was performed under the following optimized conditions: mobile phase, water containing 0.1% formic acid (solvent A) and acetonitrile (solvent B); gradient program, 20% B at 0 min, 70% B at 10 min, and 100% B at 15 min; flow rate, 1 mL/min; and detection, 306 nm.

### 2.6. Statistical Analysis

All experimental data were obtained from at least three independent experiments, and data is presented as the mean ± SD. Statistical analyses were performed using R software (version 4.3.0) with the DescTools package (version 0.99.59) [29,30]. Statistical significance was assessed using Dunnett’s multiple comparison test. For inducer-dependent comparisons at each time point, the no-inducer group at the same time point was used as the control. For time-course analyses, the 0 h sample without inducer served as the baseline control. These control definitions were applied consistently throughout the study.

## 3. Results

### 3.1. Construction of E. coli Strains for Biosensors and Biotransformation

**Figure 1:**
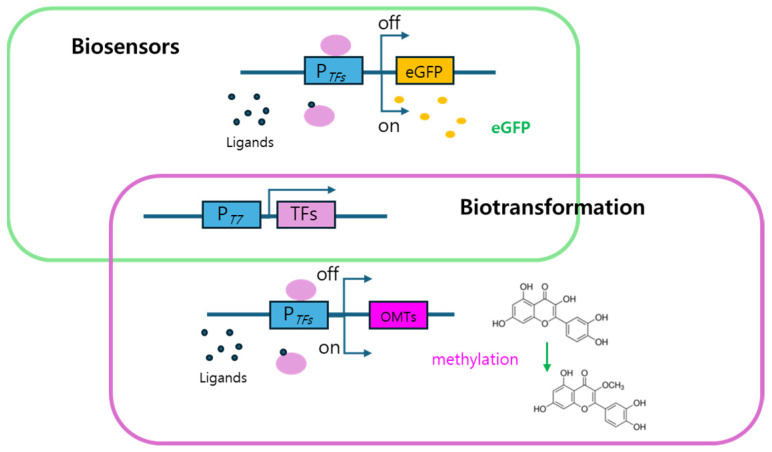
Genetic components for biosensors and *Escherichia coli* strains for biotransformation. The biosensors consisted of reporter and ligand sensing domain. For biotransformation, the eGFP in reporter domain was replaced with OMTs and the activities of OMTs confirmed the capacity of TF-based systems as gene regulatory systems. OMT, O-methyltransferase; TF, transcription factor.

To validate the potential of TF-based systems for gene regulation, we first verified that the systems selectively responded to specific ligands using *E. coli* cell-based biosensor systems. The biosensors consisted of a reporter domain producing eGFP signals and a sensing domain consisting of TFs recognizing ligands. In contrast, the *E. coli* strains used for biotransformation were constructed by replacing the reporter gene (eGFP) with O-methyltransferase (OMT) genes. The overall genetic architecture of these systems was comparable, as both employed the same transcription factor–promoter regulatory modules, differing only in the downstream gene, either the reporter or the enzyme. As illustrated in Figure 1, both systems shared identical TF–promoter pairs that functioned as ligand-responsive regulatory elements controlling gene expression in response to specific inducers. Although these systems were similar, it would be necessary to validate the functions of TF systems because their expression or regulation is different from that of genes. In the following section, we investigated the ligand-inducible gene regulation based on TFs with eGFP and diverse OMTs.

### 3.2. Validation of Expression Levels Under TF Systems

We evaluated the transcriptional regulatory efficiency of MarR and TtgR using a biosensor assay. As mentioned above, the response of biosensors based on TFs were determined by diverse factors, such as the promoter strength for both TFs and reporters, ligand selectivity and specificity, and DNA affinity of TFs. The effects of ligand selectivity and specificity of TFs were validated without modulating promoter strength. TFs were located under the T7 promoter and reporters were under the corresponding promoters for operons.

**Figure 2:**
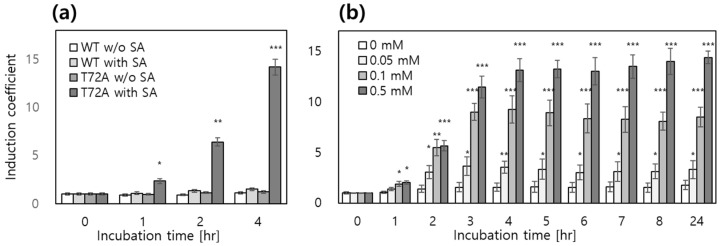
The responses of biosensors with MarR variants as regulatory proteins to SA treatment. (**a**) The biosensors harboring pMar-eGFP/pCDF-MarR WT or pCDF-MarR T72A were exposed to 0.5 mM SA, and the eGFP signals were subsequently monitored for 4 h. (**b**) The concentration dependent responses of biosensor-MarR T72A were exposed to different concentrations of SA over time. The data were obtained from three independent experiments, and the values are presented as means ± SD. Asterisks indicate significant differences in data compared with control using by Dunnett’s test (* *p* ≤ 0.05, ** *p* ≤ 0.01, *** *p* ≤ 0.001).

In the case of MarR, the expression levels of eGFP were compared in the presence of 0.5 mM SA using two MarR variants, wild-type (WT) and T72A. In previous studies, the biosensor employing MarR WT exhibited concentration-dependent responses to SA within the range of 0–2 mM, reaching an induction coefficient of approximately 2.5 at 1 mM. In contrast, the T72A variant demonstrated enhanced responsiveness to SA while retaining its repressor function [24,26]. Consistent with these findings, our biosensor assays showed that MarR WT displayed an induction coefficient value of about 1.5 to 0.5 mM of SA after 2 h of exposure, whereas the T72A variant induced markedly higher eGFP expression upon SA treatment (Figure 2a). For this reason, a full dose–response analysis of MarR WT was not included in the present study, as its limited sensitivity to SA and the superior responsiveness of the T72A variant has already been well established. These results clearly demonstrate that genetic engineering of the transcription factor enhances biosensor performance. Specifically, the engineered MarR T72A variant improved both the dynamic range and inducibility of the SA-responsive system. This provides strong evidence supporting the feasibility of employing MarR-based platforms as reliable ligand-inducible systems for gene regulation.

**Figure 3:**
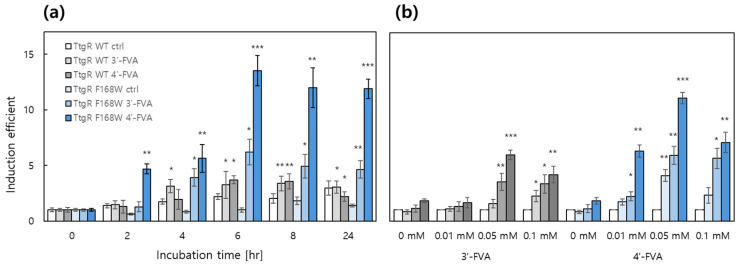
The expression levels of eGFP in biosensors using *ttg* operon systems. (**a**) The expression levels of eGFP from the biosensors harboring TtgR WT and F168W induced by 0.1 mM of 3′- and 4′-hydroxyflavanone over time. (**b**) The expression levels of eGFP induced by 0.01, 0.05, and 1 mM of 3′- and 4′-hydroxyflavanone at 0, 2, 4, and 8 h of incubation. The data was collected from three independent experiments, and values were indicated as means ± SD. Asterisks indicate significant differences in data compared with control using by Dunnett’s test (* *p* ≤ 0.05, ** *p* ≤ 0.01, *** *p* ≤ 0.001).

Unlike MarR-based biosensors, TtgR-based biosensors respond to a broader range of ligands, including resveratrol, flavones, and flavanones. A previous study reported that a biosensor harboring the TtgR F168W variant exhibited enhanced responses to flavonoids and resveratrol compared with those with the WT TtgR [25]. Among the tested flavonoids, 3′- and 4′-hydroxyflavanone (FVA) strongly induced eGFP expression; therefore, these two compounds were selected as inducers for the TtgR-based system. To validate the previous findings, biosensors containing TtgR WT or TtgR F168W were exposed to each ligand (0.1 mM), and eGFP expression was monitored over time. As shown in Figure 3a, the biosensor carrying TtgR F168W exhibited a stronger response to mono-hydroxyl-substituted flavanones, with 4′-FVA emerging as the most effective inducer.

Therefore, *E. coli* harboring pTtg-eGFP/pCDF-TtgR F168W was selected as the candidate system for gene regulation. As shown in Figure 3b, the expression levels of eGFP in this biosensor were measured over time following treatment with 0.01, 0.05, and 0.10 mM of 3′- and 4′-FVA. The *ttg* operon system repressed basal expression levels to approximately 1300 arbitrary units, whereas the repression was relieved upon the addition of an inducer. Furthermore, 4′-FVA induced a stronger eGFP expression than that with 3′-FVA, with maximum responses observed at 0.05 mM after 8 h of incubation. Although the expression levels did not exhibit strict time-dependent regulation, eGFP expression remained stable for up to 24 h. These findings indicated that the *ttg* operon-based system functions as a reliable platform for inducible gene regulation.

**Figure 4:**
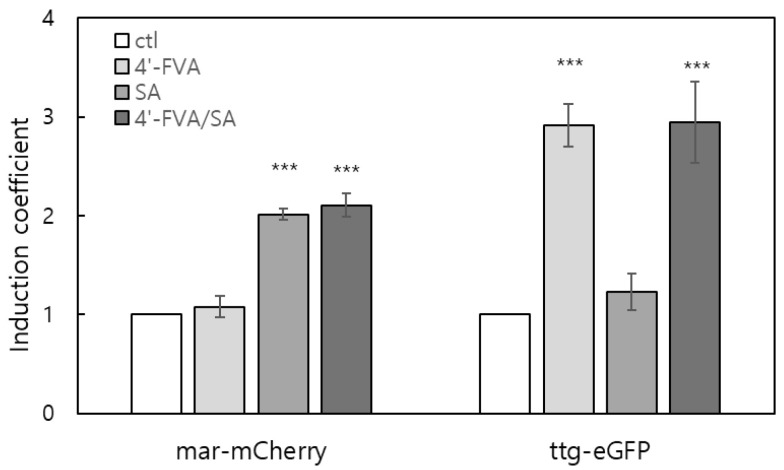
Differential expression of *mCherry* and *eGFP* under the control of MarR T72A and TtgR F168W, respectively. *E. coli* cells co-harboring reporter and regulatory constructs (P*_marR_::mCherry*/P*_ttgABC_::eGFP*, and *marR* T72A/*ttgR* F168W) were exposed to 0.5 mM salicylic acid (SA) and/or 0.05 mM 4′-hydroxyflavanone (4′-FVA) for 4 h. *mCherry* fluorescence represents the response to SA, whereas *eGFP* fluorescence indicates the response to 4′-FVA. The data was collected from three independent experiments, and values were indicated as means ± SD, respectively. Asterisks indicate significant differences in data compared with no inducer using by Dunnett’s test (*** *p* ≤ 0.001).

In addition, we examined the orthogonal ligand responses of the MarR- and TtgR-based systems to further demonstrate their applicability in metabolic engineering and synthetic biology. For this purpose, we constructed an *E. coli* strain carrying *P_marR_::mcherry*/*P_ttgABC_::egfp* along with the corresponding regulatory proteins MarR T72A and TtgR F168W. The cells were exposed to 0.5 mM SA and 0.05 mM 4′-FVA, either individually or in combination. As shown in Figure 4, each TF-based system responded exclusively to its cognate ligand, confirming clear orthogonality between the two regulatory modules. These results indicate that MarR- and TtgR-based inducible systems can operate independently within the same host, highlighting their potential as modular and orthogonal components for engineering multi-layered or sequentially regulated pathways.

Collectively, our results demonstrate that the metabolite-responsive mar and ttg operons represent novel ligand-inducible gene regulation systems with strong potential for the differential control of gene expression in response to their respective ligands. Together with the confirmed orthogonality of MarR- and TtgR-based regulation, these systems offer a promising foundation for constructing modular, multi-layered, and metabolically responsive expression platforms applicable to metabolic engineering and synthetic biology.

### 3.3. Biotransformation of Flavonoids by OMTs

Before assessing their potential as new gene expression systems, it was necessary to verify the activity of OMTs under TF-specific promoters. We first examined the methylation of different flavonoids in *E. coli* cells without the corresponding TFs. This assay was conceptually similar to biosensor analysis, but OMT expression was validated through enzymatic activities rather than reporter fluorescence. The amount of methylated products produced by OMTs over time was determined using HPLC analysis. The levels of methylated products derived from various flavonoids, including luteolin, quercetin, and apigenin, are listed in Table 2, and the structural backbones of flavonoids are shown in Figure S1. SOMT-2 under both *mar*- and *ttg*-related promoters exhibited biotransformation activities in the order of apigenin > luteolin > naringenin (Figure S2a,c). In contrast, ROMT-9 displayed the highest activity toward quercetin and luteolin (Figure S2b,d). These results are consistent with those of previous studies characterizing OMT substrate specificity [27,28]. Although quercetin was identified as the most favorable substrate for ROMT-9, neither quercetin nor its methylated products were detected after 24 h of incubation. Despite this limitation, the findings validated that OMTs expressed under the *mar* and *ttg* promoters retained their full functionality as methyltransferases, thereby indicating their feasibility for further application. Accordingly, flavonoids efficiently methylated by SOMT-2 and ROMT-9 were selected as substrates for subsequent experiments to validate the potential of these metabolite-responsive systems as novel platforms for ligand-induced gene regulation.

### 3.4. Validation of mar- and ttg-Operon Systems for New Gene Regulating Platforms

To validate the potential of TF-based systems as novel gene regulating platforms, the methyltransferase activities of SOMT-2 and ROMT-9 under the corresponding TF promoters were investigated in the presence of ligands. To test differential gene regulation, *E. coli* strains harboring SOMT-2 and ROMT-9 under the control of P*_marR_* and P*_ttgABC_* with the corresponding TFs, MarR T72A and TtgR F168W, were used in the biotransformation assay. The cells were treated with 0.5 mM of SA and 0.05 mM of 4′-FVA for the mar- and ttg-systems, respectively, and then exposed to substrates. Biotransformation efficiency was measured using HPLC analysis after 8 h of incubation, and the areas of the product peaks in the presence of inducers were compared.

**Figure 5:**
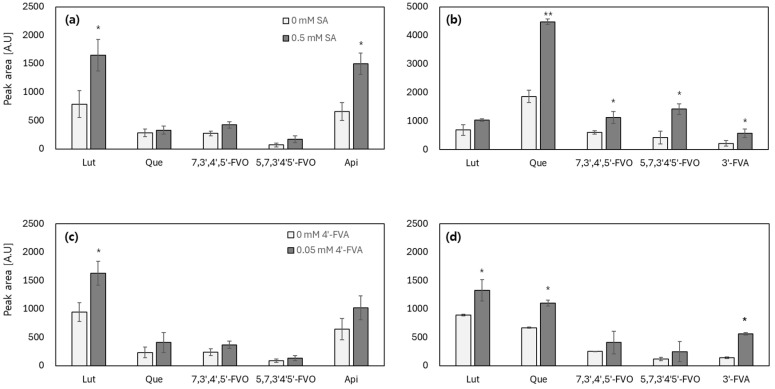
Efficiency of flavonoid methylation by OMTs under the control of mar and ttg operons with their corresponding TFs. (**a**,**b**) Biotransformation efficiency of *Escherichia coli* harboring pMar-SOMT2 and pMar-ROMT9, respectively, regulated by MarR T72A in the presence of 0.5 mM salicylic acid (SA). (**c**,**d**) Biotransformation efficiency of *E. coli* harboring pTtg-SOMT2 and pTtg-ROMT9, respectively, regulated by TtgR F168W in the presence of 0.05 mM 4′-hydroxyflavanone (4′-FVA). The *y*-axis represents the integrated peak area of methylated products quantified by HPLC and is presented as arbitrary units (a.u.). The data was collected from five independent experiments, and the values were indicated as means ± SD. Asterisks indicate significant differences in data compared with no inducer using by Dunnett’s test (* *p* ≤ 0.05, ** *p* ≤ 0.01).

When SOMT-2 and ROMT-9 were expressed under the control of the MarR- and TtgR-regulated promoters, both enzymes retained their catalytic activity and successfully produced methylated derivatives of several flavonoid substrates (Figure 5). In the mar-based system, induction with 0.5 mM SA markedly increased product formation. Specifically, SOMT-2 showed the highest activity toward luteolin and apigenin (Figure 5a), whereas ROMT-9 displayed a strong preference for quercetin, producing the largest product peaks among the tested substrates (Figure 5b). These results are consistent with previous characterizations of substrate specificity, indicating that regulation under TF-based promoters does not compromise enzymatic function.

In contrast, the ttg-based system induced by 0.05 mM 4′-FVA exhibited a more moderate induction profile (Figure 5c,d). SOMT-2 activity against luteolin and apigenin increased upon induction, although the fold-change was smaller than that of the mar-based system. Similarly, ROMT-9 was induced by quercetin, but the overall enhancement was less pronounced than that observed under the MarR control. In both systems, induction generally enhanced product yields, although the extent varied depending on the substrate. Notably, although the eGFP reporter assays demonstrated clear differences in the induction strength between the MarR and TtgR systems, the differences in biotransformation efficiency were less distinct. For example, SOMT-2-induced product formation from luteolin was only modestly higher than that of the control, suggesting that the basal expression of OMTs may substantially contribute to activity, even without ligand treatment. This pattern was particularly evident in the ttg-based system, in which the relative increase upon induction was limited.

Collectively, these findings confirm that both mar- and ttg-operon systems can regulate the expression of functional enzymes, with SOMT-2 preferentially acting on luteolin and apigenin, and ROMT-9 acting on quercetin. However, the magnitude of induction was smaller than anticipated, highlighting potential influences, such as leaky expression or substrate availability. Further interpretation of these observations, including the possible metabolic degradation of quercetin and its products during extended incubation, is outlined in the following section.

### 3.5. Application of TF-Based Systems for Flavonoid Biotransformation

**Figure 6:**
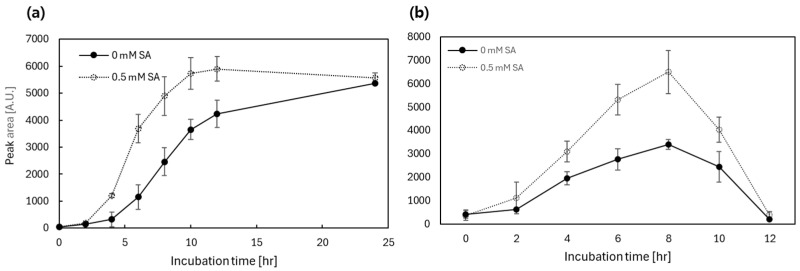
The efficiency of biotransformation by SOMT-2 and ROMT-9 under mar-operon systems with SA treatment. Peak areas of apigenin methylated by SOMT-2 (**a**) and quercetin methylated by ROMT-9 (**b**) under the regulation of the *marR*-operon system. Cells were analyzed every 2 h for 12 and 24 h. The *y*-axis represents the integrated peak area of methylated products quantified by HPLC and is presented as arbitrary units (a.u.). The data was obtained from three independent experiments, values were indicated as means ± SD.

**Figure 7:**
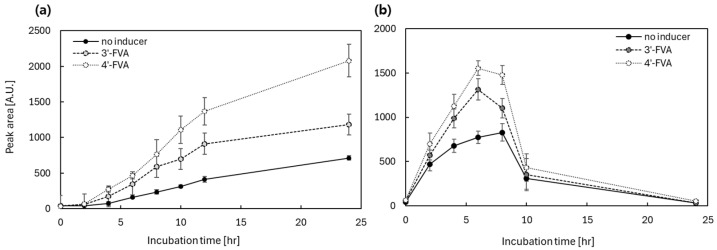
The efficiency of biotransformation by SOMT-2 and ROMT-9 under the control of the ttg-operon systems. FVA, hydroxyflavanone. Peak areas of apigenin methylated by SOMT-2 (**a**) and quercetin methylated by ROMT-9 (**b**) under the regulation of the *ttg*-operon system. The efficiency of biotransforming substrates were ordered by no-ligand treatment, 0.05 mM of 3′-FVA and 4′-FVA for both SOMT-2 and ROMT-9. The cells were analyzed every 2 h for 12 h and then after 24 h of incubation. The *y*-axis represents the integrated peak area of methylated products quantified by HPLC and is presented as arbitrary units (A.U.). The data was obtained from three independent experiments, and the values were indicated as means ± SD.

To verify the applicability of the TF-based systems, the biotransformation efficiency of flavonoids by SOMT-2 and ROMT-9, under the regulation of mar and ttg-systems, was investigated over time. Based on the results obtained above, apigenin was used as a substrate for SOMT-2 and quercetin was used for ROMT-9. To validate the *mar*-operon system, *E. coli* strains harboring *P_marR_::SOMT-2* and *P_marR_::ROMT-9* with pCDF-MarR T72A were subjected to biotransformation assays. The cells were induced with 0.5 mM SA at an OD_600_ of 0.3 with 5 mM of substrates and biotransformation efficiency was measured using HPLC analysis over time. The biotransformation efficiency was determined by calculating the peak area of the products indicated as arbitrary unit (A.U.), and results are shown in Figure 6 and Figure 7.

Endogenous *marR* is present in *E. coli*; therefore, *E. coli-marR* was used as the host cell for *mar* systems. The first point, denoted as 0 h, was the initial incubation period after substrate treatment. In the case of SOMT-2, a difference in methylated apigenin production was observed after 4 h of incubation, and the maximum differences were observed at 6 and 8 h (Figure 6a). Eventually, the biotransformation efficiency, based on the product peaks, was similar when the incubation duration was increased to 24 h. The initial expression of SOMT-2 differed with SA treatment and the amount of SOMT-2 was saturated. If a higher substrate concentration was applied, the differences would be prolonged. Additionally, ROMT-9 under *mar* systems showed similar patterns to those of SOMT-2 and an approximately two-fold increase in methylated quercetin at 8 h (Figure 6b). However, quercetin diminished after 8 h, which was consistent with the results observed during the biotransformation assay for diverse flavonoids (Figure S2).

Similar to the *mar*-operon system, apigenin and quercetin were used as substrates for SOMT-2 and ROMT-9, respectively, under the control of the *ttg*-operon system. The TF for the ttg operon, TtgR, responded differently to 3′-FVA and 4′-FVA, as shown in Figure 3; therefore, we investigated the effects of distinct inducers on flavonoids biotransformation under the control of a *ttg*-operon-based system. These results are consistent with those obtained from biosensor assays. The biotransformation efficiency by SOMT-2 and ROMT-9 were as follows: 4′-FVA > 3′-FVA > no treatment (Figure 6).

In the biotransformation assays, the production of methylated apigenin and quercetin was higher when induced with 0.05 mM of 4′-FVA than that with 3′-FVA, demonstrating that the ttg-operon system can differentially regulate gene expression depending on the strength of ligand induction. In the case of SOMT-2, enzymatic activity increased in the order of the strength of the inducers (Figure 7a). The production of methylated apigenin increased over time and cells with no inducer showed approximately five times less production. In contrast, ROMT-9 showed a similar pattern, but the quercetin levels started to diminish after 8 h of incubation. Interestingly, quercetin and its methylated derivatives diminished after approximately 8 h of incubation, whereas such disappearance was not observed for other flavonoids. Because no degradation was detected in freshly prepared standards or HPLC-only controls, this loss is presumed to result from metabolic degradation by *E. coli*, consistent with previous reports that the host bacterium can catabolize quercetin through endogenous oxidative pathways [31,32].

Based on Dunnett’s analysis, the values at 4, 6, and 8 h after 3′- and 4′-FVA exposure showed significant differences compared to the no-inducer control, whereas the differences between the 3′- and 4′-FVA treatments were not statistically significant. Nonetheless, it was noted that 4′-FVA exhibited the most pronounced increase in production efficiency, reaching approximately a 2-fold increase at 6 h. (Figure 7b). The basal (leaky) expression levels observed in the absence of ligands were relatively high, whereas the expression levels of inducible proteins were weaker than those observed in the *mar*-based system. This indicates that TtgR functions as a weaker repressor and inducer than MarR T72A. Quercetin and its methylated derivatives were no longer present after 8 h of incubation. Although the underlying mechanism remains unclear, it is likely that *E. coli* metabolized quercetin and its products during prolonged incubation.

Based on the results of both biosensor and biotransformation assays, we conclude that the MarR- and TtgR-based operon systems function as effective platforms for regulating gene expression in response to specific ligands. Their ability to respond to distinct inducers underscores their potential for differential and even sequential control of target genes. While both systems achieved clear ligand-inducible transcriptional regulation, the modulation of enzymatic activity was less pronounced, largely due to basal (leaky) expression under TF-regulated promoters. Although this limitation remains a key challenge, particularly for applications requiring stringent on/off control, it can be addressed through further optimization of TFs, promoter architecture, and associated regulatory elements. Nevertheless, the establishment of these TF-based inducible systems represents a meaningful advancement that broadens the available genetic toolkit and offers promising opportunities for metabolic engineering and synthetic biology.

## 4. Discussion

In this study, we demonstrate that metabolite-responsive TF systems derived from the *mar* and *ttg* operons can serve as ligand-inducible platforms for gene regulation in *E. coli*. By integrating flavonoid OMTs, SOMT-2, and ROMT-9, under the control of MarR- and TtgR-responsive promoters, we validated that these enzymes retained full catalytic activity and could be differentially regulated by small-molecule inducers. This expands the applications of TF-based biosensors beyond conventional reporter outputs, thereby establishing their use in the functional control of metabolic enzymes involved in biotransformation.

Biosensor assays revealed that MarR and its engineered variant (T72A) selectively responded to SA, with the T72A mutant showing enhanced induction while maintaining repressor function, consistent with previous findings that TF engineering can improve dynamic range and sensitivity [24]. Likewise, the TtgR-based system exhibited distinct responses to 4′- and 3′-FVA, demonstrating ligand-specific control and orthogonal regulation. Although this study focused on TF engineering, additional factors, such as promoter strength, TFBSs, and RBSs, are also expected to play critical roles in optimizing system performance [7,9,10]. When eGFP was replaced with OMTs, the systems successfully regulated enzymatic activities, leading to the biotransformation of flavonoids. SOMT-2 preferentially converted apigenin, luteolin, and naringenin, whereas ROMT-9 showed higher activity toward quercetin and luteolin (Figure S2). These substrate preferences were consistent with previous characterizations of OMTs, indicating that TF-based expression does not compromise enzyme specificity. Although enzyme expression levels were not directly quantified, differences in product accumulation suggested that ligand-dependent expression modulated the reaction rates rather than the final yields. Notably, quercetin and its derivatives disappeared during the biotransformation processes, likely due to catabolism by *E. coli*, emphasizing the need to consider host–substrate interactions in pathway design.

Metabolite-responsive TFs also offer distinct sensing advantages that extend beyond their role in gene regulation. Since transcription factor–ligand interactions directly regulate promoter activity, TF-based systems allow the sensitivity of the response to be tuned through engineering of TFs, TF binding sites, promoters, and ribosome-binding sequences. Their ligand-binding pockets confer high molecular specificity, which can be refined through protein engineering to discriminate among closely related metabolites. In addition, TF-based regulation enables tunable and graded responses rather than binary switching, allowing dynamic control over induction strength. These characteristics make TF-driven systems particularly attractive for biosensor development, where sensitivity, selectivity, and adjustable dynamic range are essential.

Compared with conventional inducible systems such as IPTG/LacI or arabinose/AraC, TF-based operons offer several distinct advantages. The use of orthogonal small-molecule ligands enables independent regulation of multiple genes within the same host, and graded ligand responsiveness allows differential expression levels. Such features are particularly useful in metabolic engineering, where stepwise or temporal control of pathway enzymes is often required to balance metabolic flux and prevent the accumulation of toxic intermediates [23,33,34]. In this study, the MarR T72A- and TtgR F168W-based systems successfully demonstrated ligand-dependent transcriptional regulation using both reporter assays and biotransformation experiments, highlighting the potential of metabolite-responsive TFs as modular gene-regulatory elements.

Despite these advantages, certain limitations remain. Apparently, the TF-based systems demonstrated in this study showed clear distinct responses to corresponding ligands in the levels of the fluorescent intensity from with and without ligand treatment (Figure 4). In this case, the basal expression would not be problematic, but it should be overcome to enlarge the applicability of these systems, especially for engineering the pathway related with enzymes. Despite large differences in expression levels, the corresponding differences in enzymatic activity were modest because even minimal enzyme expression produced considerable catalytic output. However, these limitations can be addressed through continued optimization of TF variants, promoter and operator sequences, and translational control elements, which will be essential for improving system stringency and expanding their practical utility. In addition, the use of exogenous inducers such as SA and 4′-FVA may raise concerns regarding potential carryover into final products. However, the concentrations used in this study were low, and typical purification steps are expected to efficiently remove these small molecules. Thus, the risk of contamination is minimal. Nonetheless, future refinements may focus on minimizing inducer amounts or developing systems activated by endogenous metabolites.

In addition to these considerations, Figure 6 and Figure 7 further reveal residual enzymatic activity in the absence of inducers, underscoring the importance of reducing background transcription. Although this leakiness limits dynamic range at the enzyme level, it also provides a clear target for future engineering. Strengthening operator affinity, stacking tandem operator sites, or improving TF–DNA binding specificity represent promising avenues for enhancing system tightness.

When compared with conventional expression systems, for example, the IPTG-inducible pET platform, the TF-based systems examined in this study are not optimized for achieving maximal production yields during biotransformation processes. Instead, the strength of these TF-based systems lies in their capacity to enable programmable, orthogonal, and potentially sequential regulation of multiple genes. Such regulatory flexibility is difficult to obtain with pET-like systems, which drive simultaneous induction of all target genes through strong promoters designed for bulk protein expression rather than conditional or differential control. Accordingly, TF-based operons may provide unique advantages in applications such as biosensing, synthetic circuit construction, and metabolic pathway engineering, where modularity, specificity, and responsiveness to metabolically relevant ligands are often more important than overall expression magnitude.

The modular nature of TF-based systems highlights their potential for diverse applications in synthetic biology and metabolic engineering. Beyond flavonoid methylation, any enzyme can be placed under TF control, enabling the programmable regulation of biocatalysis. Multi-enzyme pathways can be divided across orthogonal TFs, each triggered by a distinct ligand, enabling the sequential activation of biosynthetic modules. These strategies may be particularly useful for the production of plant-derived natural products, pharmaceuticals, and fine chemicals, for which precise temporal control is essential. Expanding the repertoire of TF-ligand pairs, combined with computational design and protein engineering, will further enhance the orthogonality and versatility of these systems.

## 5. Conclusions

The *mar* and *ttg* operon systems represent promising ligand-inducible platforms for gene regulation, beyond conventional reporter-based assays. By demonstrating selective induction and functional enzyme activity using TF-based promoters, this study provides a strong proof-of-concept for their application as programmable genetic tools. With continued optimization to reduce leakage and expand the regulatory scope, these systems have the potential to emerge as integral components of the synthetic biology toolbox, enabling orthogonal, inducible, and modular control of gene expression across diverse applications.

## Figures and Tables

**Table 1:** Lists of plasmids, *Escherichia coli* strains, and biosensors used in this study.

	Name	Description	Reference
Plasmids	pET-21a(+)	pBR322 ori, Amp^r^	Novagen
	pCDF-Duet	CloDE13 ori, Str^r^	Novagen
	pMar-eGFP	pET-21a(+) carrying P*_marR_::egfp*	[26]
	pCDF-MarRs	pCDF-Duet carrying *marR* variants	[24,25]
	pTtg-eGFP	pET-21a(+) carrying P*_ttgABC_::egfp*	[25]
	pCDF-TtgRs	pCDF-Duet carrying *ttgR* variants	[25]
	pMar-OMTs	SOMT-2 and ROMT-9 under P*_marR_*	This study
	pTtg-OMTs	SOMT-2 and ROMT-9 under P*_ttgABC_*	This study
	pMar-mCh-Ttg-eGFP	pET-Duet carrying P*_marR_::mCherry* an*d* P*_ttgABC_::egfp*	This study
	pMarR T72A-TtgR F168W	pCDF-Duet carrying *marR* T72A and *ttgR* F168W	This study
*E. coli* strains	*E. coli* BL21(DE3)	F^−^ *ompT hsdS_B_*(r_B_^−^m_B_^−^)*gal dcm lon* (DE3)	Stratagene
	*E. coli-marR*	*marR-deficient E. coli* BL21 (DE3)	[24]
Biosensor strains	biosensor-MarRs	*E. coli-marR carrying* pCDF-MarRs/pMar-eGFP	This study
	biosensor-TtgRs	*E. coli* BL21 carrying pCDF-TtgRs/pTtg-eGFP	This study

**Table 2:** List of flavonoids with position-specific substitutions of functional groups.

Abbr.	Full Name	3	5	6	7	8	2′	3′	4′	5′	6′
Lut	Luteolin	H	OH	H	OH	H	H	OH	OH	H	H
Api	Apigenin	H	OH	H	OH	H	H	H	OH	H	H
Que	Quercetin	OH	OH	H	OH	H	H	OH	OH	H	H
Kae	Kaempferol	OH	OH	H	OH	H	H	H	OH	H	H
Myr	Myricetin	OH	OH	H	OH	H	H	OH	OH	OH	H
3,5,7,3′,4′-FVO	3,5,7,3′,4′-Pentahydroxyflavone	OH	OH	H	OH	H	H	OH	OH	H	H
7,3′,4′,5′-FVO	7,3′,4′,5′-Tetrahydroxyflavone	H	H	H	OH	H	H	OH	OH	H	H
5,7,3′,4′,5′-FVO	5,7,3′,4′,5″-pentahydroxyflavone	H	OH	H	OH	H	H	OH	OH	OH	H
3′-FVO	3′-hydroxylflavone	H	H	H	H	H	H	OH	H	H	H
Dio	Diosmetin	H	OH	H	OH	H	H	OH	OCH_3_	H	H
Gos	Gossypetin	OH	OH	H	OH	OH	H	OH	OH	H	H
Nar	Naringenin	H	OH	H	OH	H	H	H	OH	H	H
3′-FVA	3′-hydroxyflavanone	H	H	H	H	H	H	OH	H	H	H
4′-FVA	4′-hydroxyflavanone	H	H	H	H	H	H	H	OH	H	H

Lut: 3′,4′,5,7-tetrahydroxyflavone; Api: 4′,5,7-trihydroxyflavone; Que: 3,3′,4′,5,7-pentahydroxyflavone; Kae: 3,4′,5,7-tetrahydroxyflavone; Myr: 3,3′,4′,5,5′,7-Hexahydroxyflavone; FVO: flavone; Dio: 3′,5,7-Trihydroxy-4′-methoxyflavone; Nar: 4′,5,7-Trihydroxyflavanone; Gos: 3,3′,4′,5,7,8-Hexahydroxyflavone; FVA: flavanone. Structural backbones of flavone and flavanone are shown in Figure S1.

## Data Availability

The original contributions of this study are included in the article/Supplementary Material. Further inquiries can be directed to the corresponding author.

## Supplementary Materials

The following supporting information can be downloaded at: https://www.mdpi.com/article/10.3390/bios15120820/s1, Figure S1: The backbone structures of the flavones and flavanones; Figure S2: Production of methylated flavonoids by OMTs under the control of TF-specific promoters, in the absence of TFs.

## Abbreviations

The following abbreviations are used in this manuscript:

LB	Lysogeny broth
OMT	O-methyltransferases
RBS	Ribosome-binding site
SA	Salicylic aid
TF	Transcription factor
TFBS	Transcription factor binding site
WT	Wild-type
