# Supplementary material for: Development of Metabolite-Responsive Transcription Factor Systems as Modular Platforms for Gene Expression Control
Source: Biosensors (Basel). 2025 Dec 18;15(12):820. doi: 10.3390/bios15120820 (PMC12730684; doi:10.3390/bios15120820)

Supplementary data for:

Development of Metabolite-Responsive Transcription Factor Systems as Modular Platforms for Gene Expression Control

Figure S1. The backbone structures of flavones and flavanones

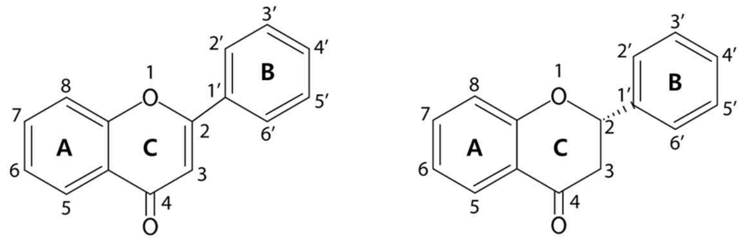

flavone flavanone

Figure S2. Production of methylated flavonoids by OMTs under transcription factor–specific promoters over time in the absence of TFs. Shown are the peak areas of methylated flavonoids produced by SOMT-2 (A) and ROMT-9 (B) under the *marR* promoter, and by SOMT-2 (C) and ROMT-9 (D) under the *ttgABC* promoter. Biotransformation efficiency was evaluated at 0, 8, and 24 h following flavonoid treatment. The y-axis represents the integrated peak area of methylated products quantified by HPLC and is presented as arbitrary units (A.U.). The data was obtained from three independent experiments, and the values were indicated as means ± SD.

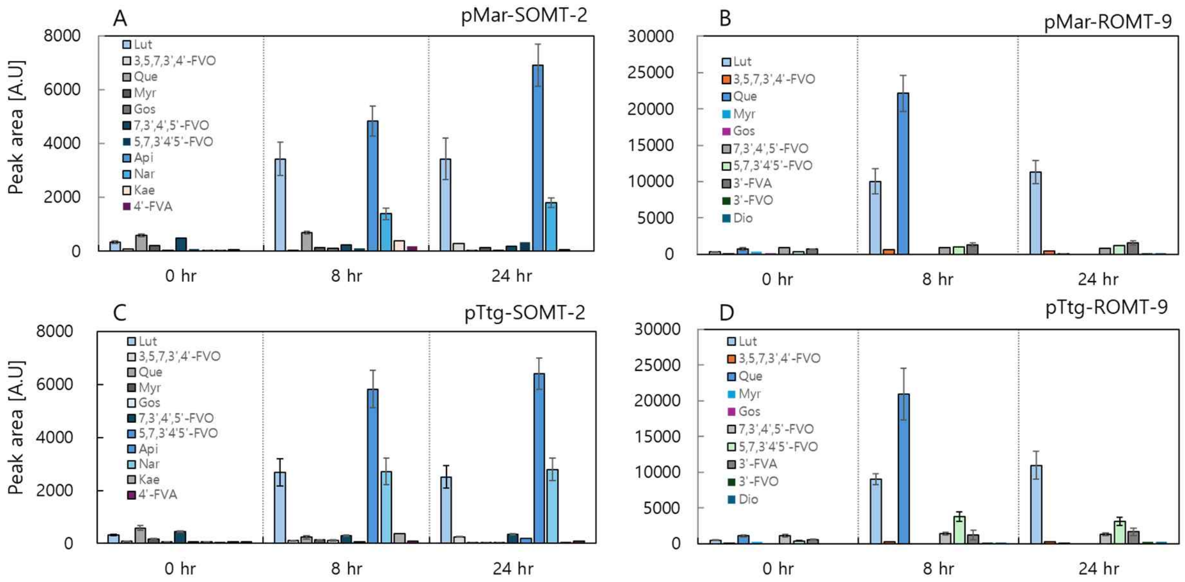

Supplement: Supplementary file 1 [file biosensors-15-00820-s001.zip › biosensors-4014107-supplementary.pdf]
